# Protocol for generating a human iPSC-derived tri-culture model to study interactions between neurons, astrocytes, and microglia

**DOI:** 10.1016/j.xpro.2025.104152

**Published:** 2025-10-22

**Authors:** Alexandra M. Lish, Paige C. Galle, Gwendolyn A. Orme, Nancy Ashour, Sarah E. Heuer, Annabel J. Curle, Christina R. Muratore, Tracy L. Young-Pearse

**Affiliations:** 1 Ann Romney Center for Neurologic Diseases, Department of Neurology, Brigham and Women’s Hospital and Harvard Medical School, Boston, MA, USA

**Keywords:** cell Biology, cell culture, Immunology, Neuroscience, stem cells, cell differentiation

## Abstract

Human induced pluripotent stem cell (hiPSC) models enable disease modeling and drug screening, but standardized methods for multi-lineage co-culture remain limited. Here, we present a cryopreservation-compatible tri-culture system of neurons, astrocytes, and microglia. We describe steps for transducing induced pluripotent stem cells (iPSCs) with cell type-specific factors, generating intermediate cryopreserved stocks, differentiating each cell population, and assembling them into tri-culture. This protocol provides a reproducible platform to study dynamic interactions between human brain cells in a physiologically relevant environment.

For complete details on the use and execution of this protocol, please refer to Lish et al.^1^^,^^2^

## Highlights

• Instructions for combining neurons, astrocytes, and microglia for brain cell modeling
• Stepwise protocol to generate and assemble human iPSC-derived tri-cultures
• Optimized media support mono-, co-, and tri-culture formats
• Adaptable timelines allow flexibility for different experimental needs

## Abstract (editor highlights)

Publisher’s note: Undertaking any experimental protocol requires adherence to local institutional guidelines for laboratory safety and ethics.

## Graphical abstract

**Figure:**
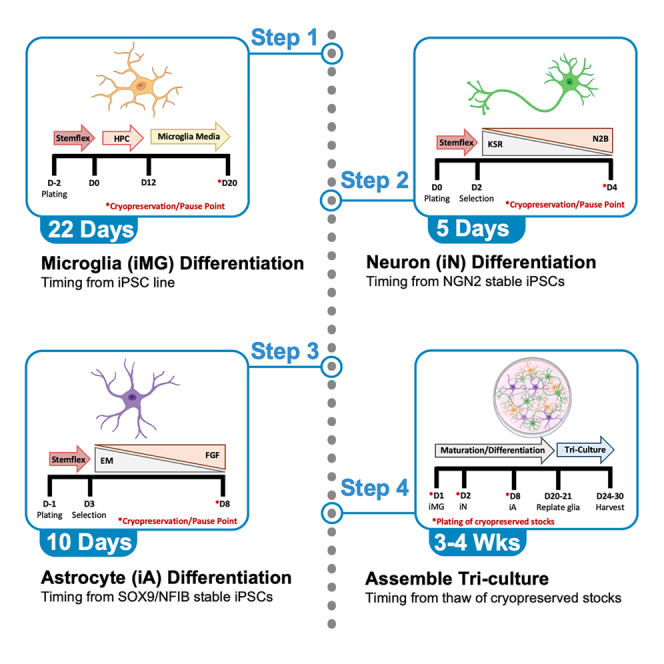

## Before you begin

Before initiating astrocyte and neuron differentiations, hiPSCs must be transduced with specific viral constructs. For neurons, TetOn-NGN2 and rtTA viruses are required, while astrocyte differentiation necessitates TetOn-Sox9, TetOn-Nfib, and rtTA viruses. Once the transduced hiPSC lines are established, cryopreserved stocks of immature neurons (Day 4), astrocytes (Day 8), and microglia (Day 20) should be prepared. Basic iPSC passaging, maintenance, and freezing skills are assumed.

In practice, there are three key stages to prepare for tri-culture.

(1) Transduction and cell banking: Establish transduced iPSC lines and generate frozen stocks of each lineage.

(2) Differentiation: Produce neurons, astrocytes, and microglia following established protocols (Zhang et al. and Lagomarsino et al.^3^^,^^4^ for neurons, Canals et al. and Lee et al.^5^^,^^6^ for astrocytes, and Abud et al., McQuade et al., and Chou et al.^7^^,^^8^^,^^9^ for microglia).

(3) Assembly of tri-culture: Thaw cryopreserved cells and combine them for co-culture.

Although the induced neuron (iN), astrocyte (iA), and microglia (iMG) differentiation methods are not unique to this protocol, we included detailed steps here for completeness and to facilitate reproducibility. If one is already experienced with generating and banking these cell types, you may skip directly to the tri-culture generation section.

Before using any cryopreserved cell stock in downstream tri-culture assembly, a vial should be thawed and plated in a test culture to assess differentiation efficiency and cellular identity. Perform immunocytochemistry (ICC) at each differentiation endpoint to ensure differentiation efficiency exceeds 95% and that there is no evidence of a contaminating proliferative population. For neurons, use antibodies against NeuN and βIII-tubulin (Tuj1); for astrocytes, use GFAP and CD44; and for microglia, use IBA1 and P2RY12. Proliferative contamination can be assessed with Ki67 staining.

***Note:*** Cell counts in this protocol were performed using a Countess II automated cell counter. Yield estimates may vary depending on the counting method or instrument. For example, we have found discrepancies between counts using the Countess II and Countess III, particularly for astrocytes, due to their larger size.

***Optional:*** For all protocols outlined below, Matrigel-coated plates can be prepared ahead of time. If not using the same day, add PBS to prevent evaporation, seal the plates with parafilm, and store at 4°C for up to 10 days.

### Innovation

This protocol describes a cryopreservation-compatible human iPSC-derived tri-culture system of neurons, astrocytes, and microglia. The individual differentiation protocols for each lineage are well established and followed as previously described. The innovation here is in the integration: we identify a single media formulation that supports all three cell types and define a plating and assembly strategy that brings them together in a stable and reproducible tri-culture. Unlike prior approaches that rely on simultaneous differentiation, which often leads to variable cell ratios and timing, our method uses cryopreserved stocks of immature neurons, astrocytes, and microglia to synchronize their introduction into co-culture. This ensures consistency across experiments and facilitates long-term storage and scalability. Together, these advances provide a practical and reproducible workflow for studying interactions among three major brain cell types, enabling broad applications in neurobiology, disease modeling, and drug discovery.

**Figure 1:**
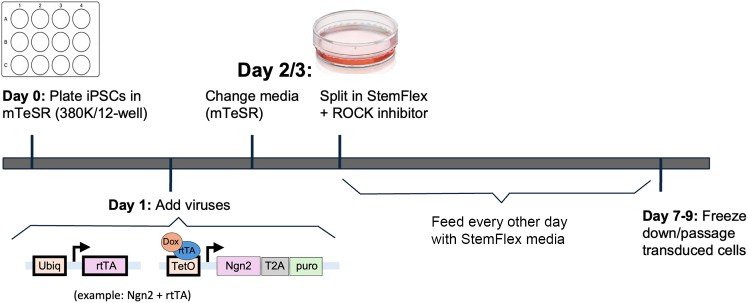
Viral transduction of iPSCs to generate inducible neurons and astrocytes Schematic overview of lentiviral transduction workflow for generating inducible iPSC lines. iPSCs are transduced with Tet-On NGN2 and rtTA constructs to produce NGN2-inducible neurons or with Tet-On SOX9, Tet-On NFIB, and rtTA constructs to produce SOX9/NFIB-inducible astrocytes. Successfully transduced iPSC lines are expanded, validated, and cryopreserved for subsequent differentiation protocols.

### Viral transduction of iPSCs to make NGN2 inducible neuron iPSCs or SOX9 and NFIB inducible astrocyte iPSCs

**Timing: 1–2 weeks** (refer to Figure 1)

1. **Day 0:** Prepare growth-factor-reduced (GFR) Matrigel-coated plates (use at 8.7 μg/cm^2^).
  a. Aliquot the appropriate volume of **cold** DMEM/F12 (e.g., 1 mL per well of a 12-well plate).
  b. Retrieve Matrigel aliquot from the −20°C freezer.
  c. Resuspend the frozen Matrigel into 1 mL **cold** DMEM/F12 by triturating gently, and combine with the remaining volume of **cold** DMEM/F12 (e.g., 12 mL total for 1 x 12 well-plate).
  d. Filter the solution through a 40 μm cell trainer.
  e. Coat one well of a 12-well plate per line to be transduced (1 mL/well).
  f. Incubate plates at 37°C for at least 30 min.
  g. Wash once with PBS before plating cells.

***Note:*** Matrigel polymerizes upon warming. Always use cold DMEM/F12 and minimize the time the Matrigel aliquot is exposed to room temperature (20°C–22°C) after removal from the freezer.

2. **Day 0:** Plate iPSCs for transduction.
  a. Plate iPSCs into one well of a 12-well plate at 380,000 cells per well (∼100,000 cells/cm^2^) in mTeSR media supplemented with 10 μM ROCK inhibitor (Y-27632, ROCKi).
  b. Incubate cells at 37°C.

***Note:*** For optimal viability, transduce iPSCs after at least one passage post-thaw rather than immediately after thawing. When replating for transduction, dissociate cells with Accutase and triturate thoroughly to prevent clumping, which can impair transduction efficiency. If non-hiPSC cell types are present, use an hiPSC selection agent such as ReLeSR, and passage again before replating with Accutase. Use low passage iPSCs to maintain consistency and cell quality. We recommend culturing cells in mTeSR prior to and during viral transduction, as StemFlex increases cell death and reduces transduction efficiency. StemFlex can be used during the passage following transduction. Basic iPSC passaging, maintenance, and freezing skills are assumed.

3. **Day 1:** Transduce iPSCs.
  a. Retrieve lentivirus from the −80°C freezer and keep on ice.
  b. Prepare 20% bleach solution.
  c. Prepare a virus master mix according to the concentrations outlined in Table 1:
    i. For each well of a 12-well plate, add the indicated amount of virus to 1 mL of mTeSR media (assuming an ultrapure titer > 10^9^).

**Table 1:** Example titer calculations for neuron and astrocyte viruses

Virus	Titer	Concentration	Volume
pTet-O-NGN2-puro lentivirus	2.97 × 10^9^	0.132 μL/50K cells	1.0
Fudelta GW-rtTA lentivirus	1.34 × 10^9^	0.132 μL/50K cells	1.0
pTet-O-SOX9-puro lentivirus	2.36 × 10^9^	0.132 μL/50K cells	1.0
pTet-O-NFIB-hygro lentivirus	3.76 × 10^9^	0.132 μL/50K cells	1.0
  d. Perform a full media change using the virus-containing media.
  e. Bleach all tips and containers that come into contact with virus.

**CRITICAL:** Lentiviral work must be performed in a certified biosafety cabinet under Biosafety Level 2 (BSL-2) conditions. Institutional biosafety regulations may vary by country or facility, and all users should follow the specific guidelines of their institution.

***Note:*** Cells should be 70%–80% confluent and form a complete monolayer for optimal transduction. This ensures an appropriate and consistent virus-to-cell ratio across cell lines and supports uniform construct expression upon induction. If cells are too sparse, feed with mTeSR and reassess the following day. If confluency remains below 70%, delay transduction and replate to achieve sufficient density for optimal yield.

***Note:*** Lentiviral titers (purchased from Alstem) typically range from 3–4 × 10^9^ for astrocyte viruses and 4–6 × 10^9^ for neuron viruses. In this protocol, 1 μL of virus is used per well, based on this approximate titer range. Since 380,000 cells are plated per well and transduction occurs after one day of growth, the virus volume per cell is an estimate and may require optimization based on actual titer and transduction efficiency. Avoid repeated freeze–thaw cycles of the virus stock.

4. **Day 2 (or Day 3):** Once confluent, split (using Accutase) each well of the 12-well plate onto one 10 cm Matrigel-coated plate.

***Note:*** At this point, cells can either be switched to StemFlex and fed on the 3–4 day flex feeding schedule (per manufacturer’s instructions) or maintained with daily mTeSR feeding.

**CRITICAL:** Regardless of confluency on Day 2, a media change must be performed—either as part of passaging or standard maintenance. Collect all media into a conical tube and bleach before discarding, as active virus may still be present. If cells are <50% confluent on Day 3, consider transferring them to a 6-well plate to support growth.

5. Feed with StemFlex (flex schedule) or mTeSR (daily).
  a. When cells reach confluency in the 10 cm plate (typically around Day 6–7), split for maintenance and expansion.
  b. Plate 2 × 10^6^ cells per 10 cm Matrigel-coated plate for continued maintenance.

**Pause point:** Expand and freeze cells after transduction but before expanding and banking neurons and astrocytes.

**CRITICAL:** It is critical to establish a stock of transduced cells early to avoid the time and cost of repeating the transduction. Freeze cells at low passage numbers, as transduced lines do not differentiate well beyond Passage 10.

**Figure 2:**
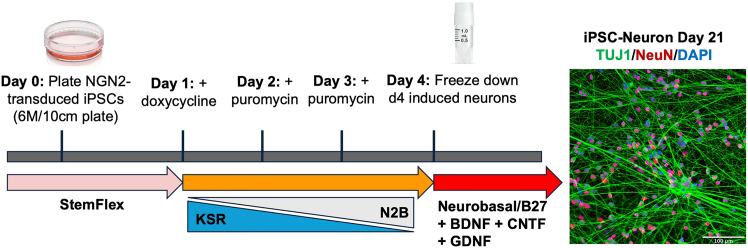
Timeline of NGN2-induced neuron differentiation from transduced iPSCs Schematic showing the four-day differentiation protocol for generating Day 4 (D4) induced neurons. NGN2-transduced iPSCs are plated on Day 0 in StemFlex medium. On Day 1, doxycycline is added to induce NGN2 expression. Puromycin selection is initiated on Days 2 and 3 to enrich for transduced cells. The culture medium is gradually transitioned from Knockout Serum Replacement (KSR) to N2B medium, followed by Neurobasal medium supplemented with B27, brain-derived neurotrophic factor (BDNF), ciliary neurotrophic factor (CNTF), and glial cell-derived neurotrophic factor (GDNF). On Day 4, D4 neurons are harvested and cryopreserved for downstream applications. Before using a cryopreserved stock, we recommend performing a test plating and differentiating cells to Day 21 to confirm appropriate morphology and expression of cell type–specific markers. Shown is a representative image of mature iPSC-derived neurons at Day 21, stained for TUJ1 (green), NeuN (red), and DAPI (blue). Scale bar = 100 μM.

### Generating and banking D4 induced neurons from NGN2 iPSCs

**Timing: 5 days** (refer to Figure 2)

6. Maintain NGN2 iPSCs at 2 × 10^6^ cells/10 cm plate (∼100,000 cells/cm^2^).
  a. Feed each 10 cm plate with 7 mL of media.
  b. If using mTeSR, feed daily and split approximately every 7 days.
  c. If using StemFlex, feed and split according to flex schedule.
7. **Day 0:** Once confluent, split (with Accutase).
  a. Plate iPSCs at 4 × 10^6^ cells per 10 cm plate (200,000 cells/cm^2^) in mTeSR (or StemFlex) media supplemented with 10 μM ROCKi.

**CRITICAL:** Do not proceed to Step 8 (Day 1) until cells reach ∼75% confluency. Maintain cells in mTeSR or StemFlex at Day 0. Cells should not be held at Day 0 for more than one day, as a dense monolayer is critical for successful differentiation. If cells are not ∼75% confluent after an additional day, re-plate and try again. If your cell line shows significant cell death after plating, consider increasing the plating density to 6 × 10^6^ cells per 10 cm plate (300,000 cells/cm^2^) to achieve the appropriate confluency.

8. **Day 1:** Feed cells with KSR media supplemented with 2 μg/mL doxycycline to induce *NGN2* expression.
9. **Day 2:** Feed cells with 1:1 mixture of KSR and N2B media supplemented with 5 μg/mL puromycin and 2 μg/mL doxycycline.

***Note:*** Puromycin concentration may need optimization depending on the cell line. We have tested a range of 1–10 μg/mL. Cells that do not express *NGN2* and the puromycin resistance gene should die during this selection step.

10. **Day 3:** Feed cells with N2B media supplemented with 1:100 B27, 5 μg/mL puromycin, and 2 μg/mL doxycycline.
11. **Day 4:** Freeze D4 neurons.
  a. Remove conditioned media.
  b. Wash the 10 cm plate with 3–4 mL PBS.
  c. Add 3 mL room temperature (20°C–22°C) diluted Accutase solution per plate (1:3 Accutase: PBS + 10 μM ROCKi).
    ***Note:*** Do not warm Accutase at 37°C, as this will inactivate the enzyme.
  d. Incubate cells at 37°C for 3–5 min, or until cells lift off the plate.
  e. Collect cells:
    i. Wash the plate with PBS to recover all remaining cells.
  f. Count cells using an automated cell counter or hemocytometer.
  g. Centrifuge at 220–300 × *g* for 5 min.
  h. Resuspend the pellet in 1 mL D4 iN media (NBM + 1:100 B27 + 5 μg/mL puromycin + 2 μg/mL doxycycline).
  i. Aliquot 1–2 × 10^6^ cells per cryovial in 0.5 mL of a 1:1 mixture of D4 iN media and freezing media (FBS + 20% DMSO).
  j. Place cryovials in a CoolCell (or equivalent) at −80°C overnight (∼24 h), then transfer to liquid nitrogen (∼−150°C for long-term storage).
    ***Note:*** BDNF, CNTF, and GDNF growth factors are not necessary in the freezing media.
    ***Note:*** Expected yield is approximately 6–12 million cells per 10 cm plate, though this may vary by cell line. Cell viability at day 4 typically ranges from 70%–100%; we do not recommend using cells with viability below 50% at the time of harvest.

**Figure 3:**
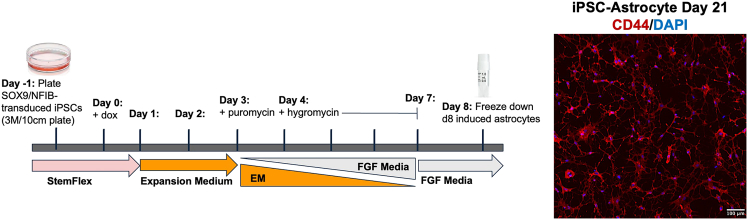
Timeline of SOX9/NFIB-induced astrocyte differentiation from transduced iPSCs Schematic showing the eight-day differentiation protocol for generating Day 8 (D8) induced astrocytes. SOX9- and NFIB-transduced iPSCs are plated on Day −1 in StemFlex medium. On Day 0, doxycycline (dox) is added to initiate transgene expression. Cells are transitioned gradually from Expansion Medium (EM) to fibroblast growth factor (FGF) Media between Days 1 and 7. Puromycin selection is initiated on Day 3, followed by hygromycin selection beginning on Day 4 and continuing through Day 7. Cultures are fully transitioned to FGF Media by Day 7. On Day 8, D8 astrocytes are harvested and cryopreserved for downstream applications. Before using a cryopreserved stock, we recommend performing a test plating and differentiating cells to Day 21 to confirm appropriate morphology and expression of cell type–specific markers. Shown is a representative image of a mature iPSC-derived astrocyte at Day 21, stained for CD44 (red) and DAPI (blue). Scale bar = 100 μM.

**Figure 4:**
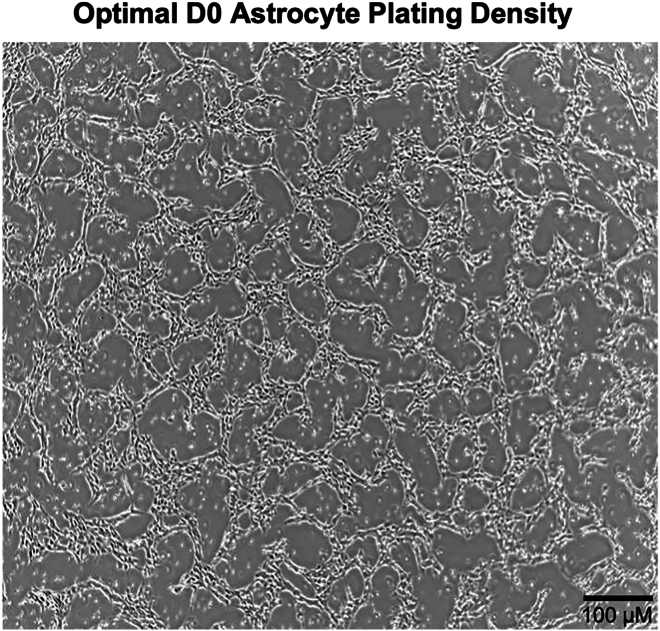
Representative image of optimal Day 0 astrocyte plating density Phase-contrast image showing the optimal single-cell plating density for initiating astrocyte differentiation on Day 0 (D0). Cells are evenly distributed across the well, with sufficient spacing to avoid overcrowding while maintaining adequate cell–cell proximity to support differentiation and survival. Scale bar = 100 μM.

### Generating and banking D8 induced astrocytes from SOX9 and NFIB iPSCs

**Timing: 10 days** (refer to Figure 3)

12. Maintain SOX9 and NFIB iPSCs.
  a. Maintain at 2 × 10^6^ cells per 10 cm plate (∼100,000 cells/cm^2^).
  b. Feed each 10 cm plate with 7 mL of media.
    i. If using mTeSR, feed daily and split approximately every 7 days.
    ii. If using StemFlex, feed and split according to flex schedule.
13. **Day -1:** Plate iPSCs for astrocyte differentiation.
  a. Once confluent, split (using Accutase).
  b. Plate iPSCs at 2–3 × 10^6^ cells per 10 cm plate (100,000–150,000 cells/cm^2^) in mTeSR (or StemFlex) media supplemented with 10 μM ROCKi.

**CRITICAL:** Seeding density may need to be optimized for each cell line. Unlike neurons, astrocytes require adequate space for proper differentiation and should not be overly crowded. Begin the differentiation process (Day 0) when the plate reaches approximately 35%–40% confluency. Refer to Figure 4 for optimal seeding density.

***Optional:*** If desired, Day −1 can be skipped, and cells can be plated directly into mTeSR (or StemFlex) media supplemented with 2.5 μg/mL doxycycline and 10 μM ROCK inhibitor to initiate Day 0. If using this approach, plate closer to 4 × 10^6^ cells per 10 cm plate (200,000 cells/cm^2^) and target a density of 50%–60% confluency. If plating with doxycycline, cells cannot be held at Day 0, as differentiation has already begun. If cell density is between 30%–60%, proceed with differentiation. If density falls outside this range, replate to achieve optimal density.

14. **Day 0:** Perform a full media change with mTeSR (or Stemflex) media supplemented with 2.5 μg/mL doxycycline.
15. **Day 1:** Perform a full media change with Expansion media supplemented with 2.5 μg/mL doxycycline.
16. **Day 2:** Perform a full media change with Expansion media supplemented with 2.5 μg/mL doxycycline.
17. **Day 3:** Perform a full media change with Expansion media supplemented with 2.5 μg/mL doxycycline and 1.25 μg/mL puromycin.

**CRITICAL:** Puromycin concentration and treatment duration must be optimized for each cell line. We have tested puromycin concentrations ranging from 1–10 μg/mL, administered from Days 3 to 7. Extending puromycin treatment beyond Day 8 is not recommended due to increased cytotoxicity and decreased cell viability. If cells become so densely packed that individual cells cannot be visualized under the microscope, additional puromycin (higher concentrations) should be added on Days 4–7. Although complete negative space is not required, clumping or piling of cells will impede further differentiation and maturation.

18. **Day 4:** Perform a full media change with a 3:1 mixture of Expansion media and FGF media, supplemented with 2.5 μg/mL doxycycline and 100 μg/mL hygromycin.

***Note:*** Hygromycin concentration may need to be optimized for each line. We have tested hygromycin concentrations ranging 100 – 200 μg/mL. However, we recommended optimizing puromycin concentration first, as puromycin acts more quickly than hygromycin, making optimization easier.

**Figure 5:**
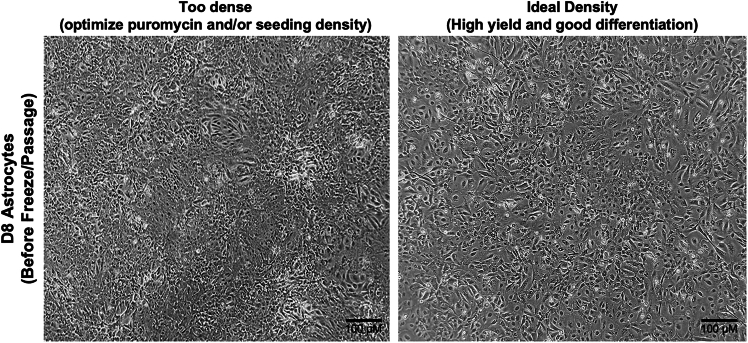
Representative images of Day 8 astrocyte cultures showing suboptimal and optimal plating densities Phase-contrast images comparing suboptimal (left) and optimal (right) Day 8 (D8) astrocyte densities prior to freeze-down or passage. Left: Cultures that are too dense show crowded morphology, which may impair further differentiation and yield. In such cases, optimization of puromycin selection and/or initial seeding density is recommended. Right: Cultures at ideal density show evenly distributed, healthy astrocytes with clear cell boundaries, supporting high yield and efficient differentiation. Scale bar = 100 μM.

19. **Day 5:** Perform a full media change with a 1:1 mixture of Expansion media and FGF media, supplemented with 2.5 μg/mL doxycycline and 100 μg/mL hygromycin.
20. **Day 6:** Perform a full media change with a 1:3 mixture of Expansion media and FGF media, supplemented with 2.5 μg/mL doxycycline and 100 μg/mL hygromycin.
21. **Day 7:** Perform a full media change with FGF media supplemented with 2.5 μg/mL doxycycline.
22. **Day 8:** Freeze D8 astrocytes.
  a. Remove conditioned media.
  b. Wash 10 cm plate with 3–4 mL PBS.
  c. Add 3 mL room temperature (20°C–22°C) diluted Accutase solution per plate (1:3 Accutase: PBS + 10 μM ROCKi).
  d. Incubate cells at 37°C for 3–5 min, or until cells have detached from the plate.
    ***Note:*** Day 8 astrocytes tend to adhere more strongly than Day 4 neurons and may require a slightly longer Accutase incubation. However, do not exceed 10 min. If detachment remains an issue, consider using a 1:1 Accutase: PBS + 10 μM ROCK inhibitor solution. However, overly dense cultures may contain less differentiated, more adherent patches that are difficult to detach. After 10 min, any remaining adherent cells should be left behind to avoid overexposure to Accutase.
  e. Collect cells:
    i. Wash the plate with PBS to recovery any remaining cells.
  f. Count cells using an automated cell counter or hemocytometer.
    ***Note:*** When using an automated cell counter, record the average cell size. Day 8 astrocytes typically have an average size of 13–15 μm. Smaller average sizes may indicate a less differentiated, more proliferative population.
  g. Centrifuge cells at 220–300 × *g* for 5 min.
    Resuspend the pellet in 1 mL of D8 iA media (FGF Media) supplemented with 2.5 μg/mL doxycycline and 10 μM ROCK inhibitor).
  h. Aliquot 1–2 × 10^6^ cells per cryovial in 0.5 mL of a 1:1 mixture of D8 iA media and freezing media (20% DMSO in FBS).
  i. Place cryovials in a CoolCell (or equivalent) at −80°C overnight (∼24 h), then transfer to liquid nitrogen (∼−150°C) for long-term storage.
    ***Note:*** Yields for Day 8 astrocytes are lower than for Day 4 neurons. Expected yield is approximately 1–3 million cells per 10 cm plate, though this may vary by cell line. Cell viability at day 8 typically ranges from 80%–100%; we do not recommend using cells with viability below 50% at the time of harvest.
    ***Note:*** If Day 8 astrocytes have an average cell size smaller than 13 μm, it does not necessarily indicate poor differentiation potential by Day 21. However, smaller, less differentiated cells are often more proliferative. To support proper maturation, plate smaller cells at a lower density on Day 8 to allow sufficient space for continued differentiation. See Figure 5 for representative examples of optimal and suboptimal day 8 densities.

**Figure 6:**
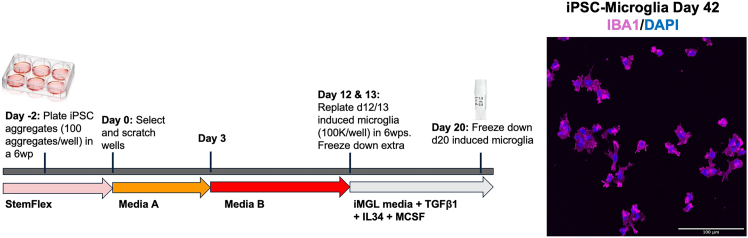
Timeline of iPSC-derived microglia differentiation and banking Schematic showing the 20-day protocol for generating and cryopreserving induced microglia from iPSCs. iPSC aggregates are plated on Day −2 in StemFlex medium. On Day 0, wells are selected and scratched to enrich for appropriate colony morphology. Media A is used from Days 0 to 2. On Day 3, a full media change is performed into Media B. Induced microglia are replated on Days 12–13 into 6-well plates (6wps) at 100,000 cells/well in iMG media supplemented with transforming growth factor beta 1 (TGF-β1), interleukin-34 (IL-34), and macrophage colony-stimulating factor (M-CSF). Cultures are maintained in this media until Day 20, when D20 induced microglia are harvested and cryopreserved for future use. For more detailed guidance on colony morphology and expected differentiation progression, refer to the STEMCELL Technologies StemDiff Hematopoietic kit documentation. Before using a cryopreserved stock, we recommend performing a test plating and differentiating cells to Day 42 to confirm appropriate morphology and expression of cell type–specific markers. Shown is a representative image of mature iPSC-derived microglia at Day 42, stained for IBA1 (magenta), and DAPI (blue). Scale bar = 100 μM.

### Generating and banking D20 microglia from iPSCs

**Timing: 22 days** (refer to Figure 6)

23. Maintain iPSCs in mTeSR or StemFlex on 6-well plates prior to starting the protocol.

***Note:*** If using cells thawed from frozen stock, it is recommended to passage once before splitting to initiate the protocol.

24. **Day -1:** Coat plates with GFR Matrigel (8.7 μg/cm^2^) as described above.

***Note:*** We recommend using 6-well plates for optimal yield, based on experience with multiple iPSC lines. However, this step can be scaled down to smaller formats (e.g., 12-well plates) or scaled up as needed, depending on experimental goals and the differentiation efficiency of the cell line.

25. **Day -1:** Dissociate iPSCs using ReLeSR.
  a. Aspirate conditioned media from the well and wash once with PBS.
  b. Add 1 mL ReLeSR per well of a 6-well plate.
  c. Incubate at room temperature (20°C–22°C) for 30 s.
  d. Aspirate ReLeSR from the well.
  e. Incubate the empty plate at 37^o^C for 2–3 min.
  f. Gently dissociate the cells by adding 1 mL of mTeSR or StemFlex supplemented with 10 μM ROCKi.

***Note:*** During dissociation, avoid vigorous mixing or agitation to preserve cell aggregates for plating.

26. **Day -1:** Plate small aggregates for differentiation.
  a. Count cells from Step 24:
    i. Add 40 μL of StemFlex media into each of two wells of a 96-well plate.
    ii. Add 5 μL of dissociated cell suspension to each well.
    iii. Visualize under a microscope and count aggregates.
    iv. Calculate the number of aggregates needed per well for plating onto 6-well plates.
  b. Plate aggregates at a density of approximately 20–30 aggregates per cm^2^.
    i. Typically, 100 – 300 aggregates are plated per well of a 6-well plate using 1.5 mL of mTeSR (or StemFlex) supplemented with 10 μM ROCKi.
  c. Gently shake the plate well to ensure uniform distribution of aggregates across the well.

**CRITICAL:** Initial plating density and even distribution are critical for successful differentiation. High density impairs mesoderm induction, while low density reduces yield. Correct density can be visually estimated by observing ∼1 aggregate per field of view (FOV) (10× objective). We recommend plating iPSCs at 2–3 different densities and selecting 2 wells on Day 0 that have optimal density to proceed with. Optimal density may vary between iPSC lines.

***Optional:*** Instead of aggregate-based counting, different split ratios can be used to achieve appropriate cell density. For example, from an ∼80% confluent 6-well plate, a split ratio between 1:500 and 1:2000 can be used for plating.

**CRITICAL:** Do not proceed with differentiation on Day 0 if the iPSC colonies are too small. If necessary, perform a full media change the following day with mTeSR or StemFlex to allow additional growth before initiating differentiation. Refer to the STEMdiff Hematopoietic Kit documentation for representative images of optimal aggregate density.

27. **Day 0:** Select wells with properly size colonies to begin hematopoietic precursor cell (HPC) differentiation according to the STEMdiff Hematopoietic Kit.
  ***Note:*** Ideally, colonies should be large but smaller than an entire 10× FOV and distributed at approximately one colony per 10× FOV.
  ***Optional:* (recommended):** To maximize surface area for the growth of well-sized colonies, carefully remove smaller excess cells and colonies using a pipette tip. This should only be performed if a microscope is available within the biosafety cabinet to allow precise visualization and sterile technique.
  a. Aspirate media from the selected wells.
  b. Wash once with PBS.
  c. Add 2 mL of Medium A per well of the 6-well plate.
    ***Note:*** For this step and the following one, warm Medium A to room temperature (20°C–22°C). Do not place Medium A in a 37°C bead bath.
28. **Day 2:** Perform a half media change with Medium A.
  a. Remove 950 μL of media from each well.
  b. Replace with 1 mL of fresh Medium A.
29. **Day 3:** Perform a full media change with Medium B.
  a. Aspirate all media from the well.
  b. Add 2 of Medium B mL per well of a 6-well plate.

***Note:*** For this step and the following one, warm Medium B to room temperature (20°C–22°C). Do not place Medium B in a 37°C bead bath.

**Figure 7:**
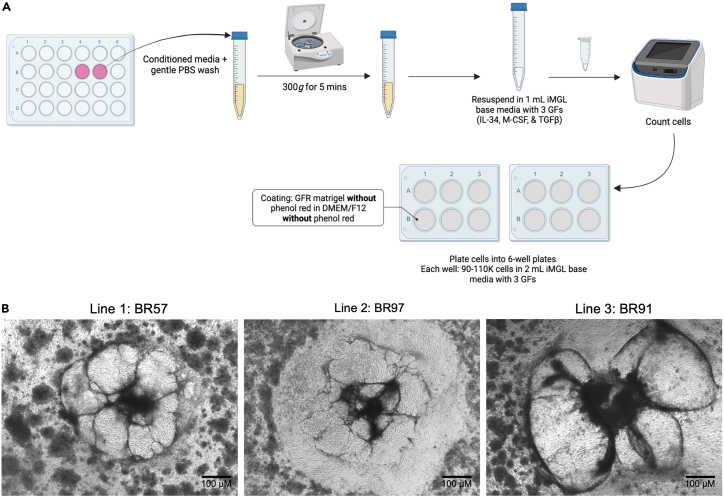
Workflow for Day 12/Day 13 collection and replating of hematopoietic progenitor cells (HPCs) during microglia differentiation (A) Schematic outlining the collection, centrifugation, counting, and replating of non-adherent HPCs from Day 12/Day 13 cultures. Conditioned media and gentle PBS washes are collected, centrifuged at 300 × g for 5 min, and resuspended in 1 mL iMG base media supplemented with interleukin-34 (IL-34), macrophage colony-stimulating factor (M-CSF), and transforming growth factor beta 1 (TGF-β1). Cells are counted and replated onto phenol red-free Matrigel-coated 6-well plates at 90,000–110,000 cells/well in 2 mL iMG base media with growth factors. (B) Representative phase-contrast images of Day 12 cultures from three independent iPSC lines (BR57, BR97, BR91), illustrating normal variation in morphology across different differentiations and genetic backgrounds. For more detailed morphological guidance, refer to the STEMCELL Technologies StemDiff Hematopoietic kit documentation. Scale bar = 100 μM.
33. **Day 12:** Plate HPCs for microglia differentiation.
  a. Plate HPCs at 100,000 cells per well onto GFR Matrigel–coated 6-well plates.
  b. Plate cells into 2 mL of iMG base media supplemented with 100 ng/mL IL-34, 25 ng/mL M-CSF, and 50 ng/mL TGF-β1 per well.

***Note:*** Do not add growth factors to the media until you have determined the total volume of media needed for plating. Unused iMG base media without growth factors can be stored at 4°C for future feedings.

***Note:*** During differentiation from HPCs to microglia, cells may grow both adherently and non-adherently.

30. **Day 5, 7, and 10:** Perform a half medium change with Medium B.
  a. Remove 950 μL of media from each well.
  b. Replace with 1 mL of fresh Medium B.
31. **Day 12:** Prepare media and Matrigel-coated plates for day 12 collection.
  ***Note:*** Microglia yield is more variable across lines compared to astrocytes and neurons. Day 12 yields typically range from 750,000 to 3,000,000 cells from two wells of a 6-well plate.
  a. Coat four 6-well plates (24 wells total) per cell line/differentiation with growth factor–reduced, phenol-red free Matrigel (1 mg/12 mL phenol-red free DMEM/F12).
    ***Note:*** Matrigel prepared with phenol red–containing medium is also acceptable.
  b. Warm 50 mL of iMG base media per cell line/differentiation.
  c. If planning to collect cells on Day 13, also warm medium B to room temperature (20°C–22°C). Prepare enough volume for 1 mL per well of a 6-well plate.
32. **Day 12:** Collect non-adherent HPCs from the culture (see Figure 7).
  a. Carefully collect all media from the wells and wash each well once with PBS to collect any remaining non-adherent cells.
    **CRITICAL:** Perform PBS wash gently to avoid dislodging adherent cells. Only non-adherent, floating cells should be collected. Combine PBS wash with the media collected in Step 31a.
  b. If planning to collect additional cells on Day 13 (recommended to maximize total yield), add 1 mL of Medium B to each well.
    ***Note:*** 1 mL is sufficient because cells will be collected again the following day.
  c. Centrifuge harvested non-adherent cells at 220–300 × *g* for 5 min to pellet the cells.
  d. Resuspend the pellet in 1 mL iMG base media supplemented with 100 ng/mL IL34, 25 ng/mL M-CSF, and 50 ng/mL TGF-β1.
    **CRITICAL:** Add growth factors fresh to the media immediately before use.
  e. Count cells. If viability is less than 30%, do not proceed.

**Pause point:** If desired, Day 12 HPCs can be cryopreserved instead of proceeding with microglia differentiation. Resuspend cells in 500 μL BamBanker per 500,000–2 million cells for freezing.

**Figure 8:**
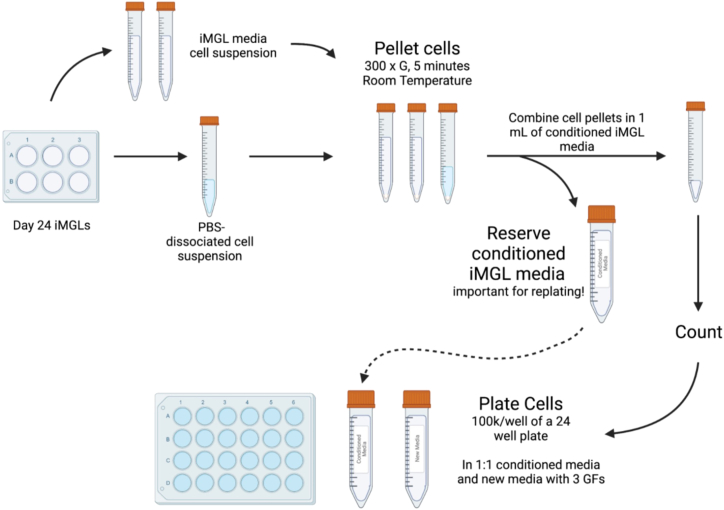
Workflow for microglia collection and replating past Day 20 Schematic outlining the collection, centrifugation, and replating workflow for induced microglia (iMGs). Conditioned media and PBS-dissociated cells are collected separately. Cells are pelleted by centrifugation at 300 × g for 5 min at room temperature (20°C–22°C), then resuspended in 1 mL conditioned iMG media. For replating applications (e.g., to address overcrowding as described in problem 2), conditioned media must be kept separate from PBS washes to allow replating in a 1:1 mixture of conditioned and fresh iMG base media supplemented with growth factors. In contrast, when preparing cells for cryopreservation, conditioned media and PBS washes can be combined, as conditioned media is no longer required for post-thaw recovery. Cells are typically replated at 100,000 cells/well of a 24-well plate.

34. **Day 13:** Replate or freeze Day 13 HPCs.
  a. Collect Day 13 HPCs and either replate or freeze them following the same procedure used for Day 12. Discard the original plate after collection.
35. **Day 14, 16, and 18:** Feed cells with fresh supplemented iMG base media.
  a. Add 1 mL fresh iMG base media supplemented with 100 ng/mL IL-34, 25 ng/mL M-CSF, and 50 ng/mL TGF-β1 per well of a 6-well plate.
    **CRITICAL:** Do not fully remove old media during microglia differentiation. Cells secrete paracrine cytokines that are important for supporting efficient differentiation.
36. **Day 20:** Freeze microglia (see Figure 8).
  a. Collect the conditioned media containing the floating cells into conical tubes.
  b. Add 1 mL of PBS to each well and incubate at room temperature (20°C–22°C) for 10–15 min.
  c. Transfer the PBS wash to the same conical tubes containing the conditioned media.
  d. Check under the microscope to confirm that all the cells have detached. If adherent cells remain, repeat Steps b-c.
  e. Centrifuge cells at 300g for 5 min.
  f. Remove the supernatant from each tube. Gently resuspend and combine the pellets in 0.5–1 mL BamBanker freezing media.
    ***Note:*** Use 0.5 mL of BamBanker freezing media if the total cell count is expected to be under 1 million. Alternatively, resuspend cells initially in iMG base media to perform counting, then centrifuge again and resuspend in BamBanker freezing media.
  g. Count the cells.
  h. Adjust the final volume of BamBanker freezing media based on the total cell count. It is recommended to freeze 0.5-2M cells per cryovial in 500 μL BamBanker.
  i. Store vials at −80^o^C for 24 h in CoolCell (or equivalent), then transfer to liquid nitrogen for long-term storage.
    ***Note:*** Expected yield is approximately 3–5 million cells for 4 × 6-well plates, though this can range from 0.5–20M depending on the line. Cell viability at day 20 typically ranges from 70%–100%; we do not recommend using cells with viability below 40% at the time of harvest. A good day 20 yield show approximately double or triple the amount plated at day 12.

## Key resources table

**Table:** 

REAGENT or RESOURCE	SOURCE	IDENTIFIER
**Antibodies**		

Chicken anti-MAP2 (1:1,000 WB and ICC)	Abcam	Cat #AB5392; RRID: AB_2138153
Rabbit anti-NeuN (1:1,000 WB, 1:100 ICC)	Abcam	Cat # ab177487. RRID: AB_2532109
Chicken anti-TUJ1 (1:2,000 WB, 1:500 ICC)	Novus	Cat# nb100-1612. RRID: AB_10000548
Goat anti-AIF1 (1:500 WB, 1:200 ICC)	Abcam	Cat# ab5076; RRID: AB_2224402
Mouse anti-CD44 (1:2,000 WB, 1:500 ICC)	Abcam	Cat# ab254530. RRID: AB_2885131
Mouse anti-GAPDH (1:10,000 WB)	Proteintech	Cat# 60004-1-Ig; RRID: AB_2107436

**Bacterial and virus strains**		

pTet-O-NGN2-puro	Zhang et al.^3^	Addgene plasmid #52047
FUdeltaGW-rtTA	Zhang et al.^3^	Addgene plasmid #19780
Tet-O-SOX9-puro	Canals et al.^5^	Addgene plasmid #117269
Tet-O-NFIB-hygro	Canals et al.^5^	Addgene plasmid #117271

**Chemicals, peptides, and recombinant proteins**		

Neurobasal medium	Life Technologies	Cat# 21103049
GlutaMAX	Life Technologies	Cat# 35050-061
MEM NEAA	Life Technologies	Cat# 11140050
Dextrose	Sigma-Aldrich	Cat# D9434-250G
STEMdiff hematopoietic kit	STEMCELL Technologies	Cat# 5310
Insulin-transferin-selenite (ITS-G)	Thermo Fisher Scientific	Cat# 41400045
Monothioglycerol	Sigma-Aldrich	Cat# M1753-100ML
Insulin	Sigma-Aldrich	Cat# I2643-50MG
StemFlex medium combo kit	Life Technologies	Cat# A3349401
StemPro Accutase	Life Technologies	Cat# A1110501
ReLeSR	STEMCELL Technologies	Cat# 100-0484, 100-0483
Matrigel (growth-factor reduced)	Corning	Cat# 354230
Matrigel (regular)	Corning	Cat# 354234
Matrigel (phenol red-free)	Corning	Cat# 356231
DMEM/F12	Thermo Fisher Scientific	Cat# 11320033
DMEM/F12 (phenol red-free)	Thermo Fisher Scientific	Cat# 11039047
Knockout DMEM	Gibco	Cat #10829018
BamBanker	Fisher Scientific	Cat# 50-999-555
DMSO	Sigma-Aldrich	Cat# D2650-100mL
FBS	Corning	Cat# 35011CV
N2 supplement	Invitrogen	Cat# 17502048
B27 supplement	Life Technologies	Cat# 17504-044
Sodium pyruvate	Thermo Fisher Scientific	Cat# 11360070
mTeSR1 Complete Kit	STEMCELL Technologies	Cat# 85850
BrainPhys neuronal medium and SM1 kit	STEMCELL Technologies	Cat# 5792
Ara-C	Sigma-Aldrich	Cat# C1768-100MG
BDNF	PeproTech	Cat# 450-02
CNTF	PeproTech	Cat# 450-13
GDNF	PeproTech	Cat# 450-10
TGF-β1	Miltenyi Biotec	Cat# 130-108-969
IL-34	PeproTech	Cat# 200-34-250UG
M-CSF	Thermo Fisher Scientific	Cat# PHC9501
CX3CL1	PeproTech	Cat# 300-31-50UG
CD200	Bon Opus	Cat# BP004-50UG
Y-27632 (Rock inhibitor)	STEMCELL Technologies	Cat# 72304
Doxycycline hyclate	Sigma-Aldrich	Cat# D9891-5g
Puromycin	Gibco	Cat# A11138-03
Penicillin-streptomycin	Thermo Fisher Scientific	Cat# 15140122
Hygromycin B Gold	InvivoGen	Cat# ant-hg-1
FGF2	PeproTech	Cat# 100-18B
BMP4	PeproTech	Cat# 120-05et-50ug
N-acetylcysteine	Sigma-Aldrich	Cat# A8199-10G
Heparin-binding EGF-like growth factor	Sigma-Aldrich	Cat# E4643-50ug
dbcAMP	Sigma-Aldrich	Cat# D0627-250mg
PBS	Invitrogen	Cat# 14190-250
Knockout replacement serum (KSR)	Invitrogen	Cat# 10828-028
Beta-mercaptoethanol	Invitrogen	Cat# 21985-023
Dextrose	Sigma	Cat #D9434-250g
N2 supplement B	STEMCELL Technologies	Cat# 07156
Laminin	Invitrogen	Cat# 23017-015
Poly-L-ornithine	Sigma	Cat# P3655

**Experimental models: Cell lines**		

BR11	Lagomarsino et al.^4^	BR11, AJ0002
BR33	Lagomarsino et al.^4^	BR33, AJ0047
BR57	Lagomarsino et al.^4^	BR57, AJ0073
BR91	Lagomarsino et al.^4^	BR91, AJ0115
BR97	Lagomarsino et al.^4^	BR97, AJ0123

**Other**		

5 mL Eppendorf microcentrifuge tubes	Sigma	Cat# EP0030119487
Anti-adherence rinsing solution	STEMCELL Technologies	Cat# 07010
10 cm plates	BD Falcon	Cat# 353003
6-well plates	Corning	Cat# 3506
12-well plates	Corning	Cat# 3512
24-well plates	Corning	Cat# 3527

## Materials and equipment

**Table:** Reagent preparation

Reagent	Reconstitution solution	Working concentration (final use)	Stock/Aliquot concentration	Aliquot volume and storage
BDNF	0.1% BSA/PBS	10 ng/mL	10 μg/mL	500 μL aliquots; store at −20°C
CNTF	0.1% BSA/PBS	10 ng/mL	10 μg/mL	500 μL aliquots; store at −20°C
GDNF	0.1% BSA/PBS	10 ng/mL	10 μg/mL	500 μL aliquots; store at −20°C
ROCK inhibitor (Y-27632)	DMSO	10 μM	10 mM	300 μL aliquots; store at −20°C
Puromycin	Pre-dissolved stock	1–10 μg/mL	10 mg/mL	1 mL aliquots; store at −20°C
Hygromycin	Pre-dissolved stock	100 μg/mL	100 mg/mL	1 mL aliquots; store at −20°C
Doxycycline hyclate	Sterile water	2 μg/mL (neurons) 2.5 μg/mL (astrocytes)	20 mg/mL	300 μL aliquots; store at −20°C
Dextrose	MilliQ water	20% solution	Powder	Store at 4°C
N2 supplement	Ready to use	1X	100X	Store at −20°C
B27 supplement	Ready to use	1X or 2X	50X	Store at −20°C
bFGF	5 mM Tris-HCl + 0.1% BSA	8 ng/mL	10 μg/mL	400 μL aliquots; store at −20°C
BMP4	0.1% BSA + 4 mM HCl	10 ng/mL	10 μg/mL	500 μL aliquots; store at −20°C
dbcAMP	Sterile water	500 μg/mL	100 mg/mL	500 μL aliquots; store at −20°C (protect from light)
N-acetylcysteine	Sterile water	5 μg/mL	50 mg/mL	30 μL aliquots; store at −20°C
HB-EGF	0.1% BSA/PBS	5 ng/mL	50 μg/mL	30 μL aliquots; store at −20°C
Monothioglycerol	PBS	1 M	11.5 M stock	Store at 4°C (foil-wrapped)
Insulin	0.01 N HCl	5 μg/mL	5 mg/mL	Sterile filtered; store at 4°C
M-CSF	20 mM Tris-HCl + PBS + 0.1% BSA	25 ng/mL	25 μg/mL	250 μL aliquots; store at −80°C
IL-34	PBS + 0.1% BSA	100 ng/mL	100 μg/mL	200 μL aliquots; store at −80°C
TGF-β1	Sterile deionized water	50 ng/mL	50 μg/mL	200 μL aliquots; store at −80°C
CX3CL1	Sterile deionized water	100 ng/mL	100 μg/mL	200 μL aliquots; store at −80°C
CD200	Sterile deionized water	100 ng/mL	100 μg/mL	200 μL aliquots; store at −80°C
Laminin	Supplied in solution	5 μg/mL	1 mg/mL	60 μL aliquots; store at −80°C
Poly-L-ornithine	PBS	100 μg/mL	50 mg/mL	100 μL aliquots; store at −80°C
Matrigel	Supplied in 10 mL bottle	Cell-type dependent	0.5 to 4 mg aliquots	Store aliquots at −20°C

**Table:** mTeSR media

Reagent	Stock concentration	Final concentration	Amount
mTeSR Medium	N/A	N/A	800 mL
mTeSR Supplement	N/A	N/A	200 mL
PenStrep	N/A	N/A	10 mL
**Total**	**N/A**	**N/A**	**1 L**

[Sterile filter and store at 4°C for up to 1 month]

**Table:** StemFlex media

Reagent	Stock concentration	Final concentration	Amount
StemFlex Medium	N/A	N/A	900 mL
StemFlex Supplement	N/A	N/A	100 mL
**Total**	**N/A**	**N/A**	**1 L**

[Sterile filter and store at 4°C for up to 1 month]

**Table:** Knockout replacement serum (KSR) media

Reagent	Stock concentration	Final concentration	Amount
Knockout DMEM	N/A	N/A	415 mL
KSR	N/A	N/A	75 mL
MEM NEAA	100×	1×	5 mL
Beta-mercaptoethanol	N/A	N/A	0.5 mL
Glutamax	100×	1×	5 mL
**Total**	**N/A**	**N/A**	**500 mL**

[Sterile filter, cover in aluminum foil, and store at 4°C for up to 1 month]

**Table:** N2B media

Reagent	Stock concentration	Final concentration	Amount
DMEM/F12	N/A	N/A	482.5 mL
Dextrose	100%	20%	7.5 mL
N2 supplement B	100×	1×	5 mL
Glutamax	100×	1×	5 mL
**Total**	**N/A**	**N/A**	**500 mL**

[Sterile filter, cover in aluminum foil, and store at 4°C for up to 1 month]

**Table:** Neurobasal medium (NBM)

Reagent	Stock concentration	Final concentration	Amount
Neurobasal Medium	N/A	N/A	485 mL
Glutamax	100×	1×	5 mL
MEM NEAA	100×	0.5×	2.5 mL
Dextrose	100%	20%	7.5 mL
**Total**	**N/A**	**N/A**	**500 mL**

[Sterile filter and store at 4°C for up to 1 month]

**Table:** Expansion media (EM)

Reagent	Stock concentration	Final concentration	Amount
DMEM/F12	N/A	N/A	440 mL
Glutamax	100×	1×	5 mL
N2 Supplement	100×	1×	5 mL
FBS	100%	10%	50 mL
**Total**	**N/A**	**N/A**	**500 mL**

[Sterile filter and store at 4°C for up to 1 month]

**Table:** Fibroblast growth factor (FGF) base media

Reagent	Stock concentration	Final concentration	Amount
Neurobasal Medium	N/A	N/A	485 mL
Glutamax	100×	1×	5 mL
MEM NEAA	100×	1×	5 mL
FBS	100%	1%	5 mL
**Total**	–	**N/A**	**500 mL**

[Sterile filter and store at 4°C for up to 1 month]

**Table:** Maturation base media (MM)

Reagent	Stock concentration	Final concentration	Amount
Neurobasal Medium	N/A	N/A	242.5 mL
DMEM/F12	N/A	N/A	242.5 mL
N2	50×	1×	5 mL
Glutamax	100×	1×	5 mL
Sodium Pyruvate	100×	1×	5 mL
**Total**	**N/A**	**N/A**	**500 mL**

[Sterile filter and store at 4°C for up to 1 month]

**Table:** Preparation of StemDiff hematopoietic differentiation media

Reagent	Component	Volume
Medium A	Basal Medium	45 mL
	Supplement A	225 μL
Medium B	Basal Medium	75 mL
	Supplement B	375 μL

[Store at 2°C–8°C for up to 3 weeks OR store at −20°C for up to 6 months]

**Table:** iMG base media

Reagent	Stock concentration	Final concentration	Amount
Phenol Red Free DMEM/F12	N/A	N/A	452 mL
Insulin-transferin-selenite (ITS-G)	100×	2×	10 mL
N2	50×	0.5×	2.5 mL
Glutamax	100×	1×	5 mL
B27	50×	2×	20 mL
NEAA	100×	1×	5 mL
Monothioglycerol	1M	400 μM	200 μL
Insulin	5 mg/mL	5 μg/mL	500 μL
PenStrep	N/A	N/A	5 mL
**Total**	**N/A**	**N/A**	**500 mL**

[Sterile filter, cover in aluminum foil, and store at 4°C for up to 1 month]

## Step-by-step method details

With cryopreserved stocks of day 4 neurons, day 8 astrocytes, and day 20 microglia prepared, the next step is to thaw these cells, resume their differentiation, and assemble them into the tri-culture system. The following sections outline how to continue microglia differentiation after the day 20 PAUSE/FREEZE point, astrocyte differentiation after the day 8 PAUSE/FREEZE point, and neuron differentiation after the day 4 PAUSE/FREEZE point. In these sections, “day” refers to the timeline beginning at the thaw of the first cryopreserved stock, as shown in Figure 11.

### Thaw and differentiate D20 iPSC microglia

**Timing: 20 days**

Here, we outline the steps for thawing and differentiating Day 20 iPSC-derived microglia in preparation for integration into the triple culture system.

1. **Day 1:** Thaw and plate day 20 cryopreserved microglia.
  a. Coat plates with growth factor-reduced (GFR) Matrigel prepared in phenol red free DMEM/F12 (e.g., 1 mg/12 mL). If plating in 24 well-plates, add 500 μL of diluted Matrigel to each well.
    ***Note:*** Phenol-red free is preferred, but Matrigel and DMEM/F12 with phenol red is also sufficient.
  b. Thaw cryovial(s) in a water or bead bath until mostly thawed.
  c. Add 0.5 mL warmed iMG base media (without growth factors) directly to each cryovial.
  d. Transfer the contents of each cryovial to a labeled conical tube.
  e. Wash each vial with 1 mL of iMG base media (without growth factors) and transfer the wash to the same conical tube.
    ***Note:*** To improve cell recovery and viability after thawing, gently invert the conical tube several times before centrifugation to evenly disperse the freezing medium. This minimizes pooling of cryoprotectant around the cell pellet and may reduce cellular stress.
  f. Centrifuge cells at 220–300 × *g* for 5 min.
  g. Carefully remove the supernatant and resuspend the pellet in 1 mL of iMG media supplemented with 100 ng/mL IL-34 + 25 ng/mL M-CSF + 50 ng/mL TGF-β1.
    **CRITICAL:** Growth factors should be added fresh to the media immediately prior to use.
  h. Count the cells.
    ***Note:*** With proper freezing and banking techniques, post-thaw viability should be comparable to, or slightly lower than, the original freeze. If a significant drop in viability is observed after thawing, assess whether the cells are suitable for continued use.
  i. Adjust the final suspension volume with iMG base media supplemented with 100 ng/mL IL-34, 25 ng/mL M-CSF, and 50 ng/mL TGF-β1) based on the cell count and desired plating density.
  j. Plate cells onto the Matrigel-coated plates.
    ***Note:*** Based on our experience, microglia exhibit optimal growth when plated at a density of ∼53,000 cells/cm^2^, corresponding to approximately 80,000–120,000 cells per well of a 24-well plate. However, plating density may vary depending on the proliferative capacity of the cell line. Use of larger plate formats can increase the proportion of non-adherent to adherent cells, leading to a greater cell loss during media changes. We recommend starting with 100,000 cells per well in a 24-well plate to optimize recovery and growth.
2. **Day 2:** Feed microglia without media removal.
  a. For each well of a 24 well plate, add 0.5 mL of fresh iMG base media supplemented with 100 ng/mL IL-34, 25 ng/mL M-CSF, and 50 ng/mL TGF-β1 directly to the existing media. Do not remove any media prior to feeding.
3. **Days 4, 6, 8, 10, 12, 14, and 16:** Perform one-third media changes.
  a. For each well of a 24-well plate, remove 450 μL of old media.
  b. Add 0.5 mL of fresh iMG base media supplemented with 100 ng/mL IL-34, 25 ng/mL M-CSF, and 50 ng/mL TGF-β1.

**Figure 9:**
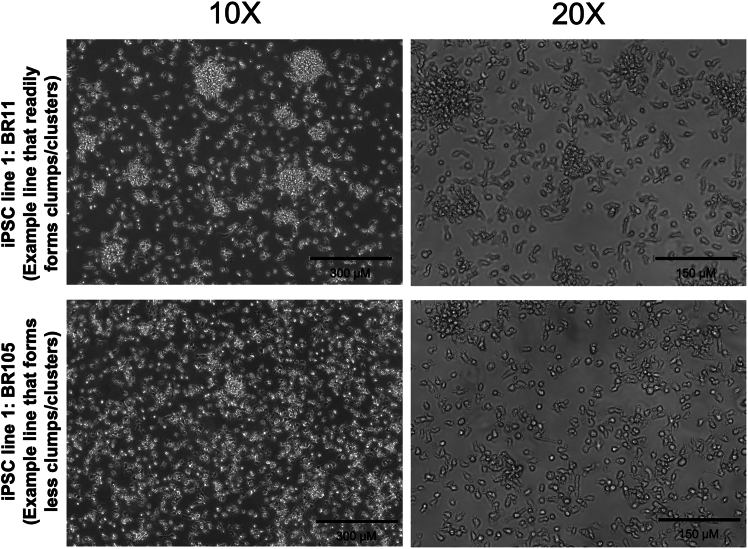
Representative images showing variability in microglia clumping across iPSC lines Phase-contrast images at 10× and 20× magnification comparing two iPSC lines during microglia differentiation. Top: iPSC line BR11, an example of a line that readily forms visible clumps and clusters during growth. Bottom: iPSC line BR105, an example of a line that forms fewer clumps and remains more evenly distributed. Although clumping does not always indicate poor cell health—both cultures maintained high viability in this example—the presence of clumps should be monitored, as in some lines excessive clustering can signal overcrowding and may lead to subsequent cell death.

**CRITICAL:** Microglia continue to proliferate throughout differentiation, and growth rates can vary between cell lines. Some lines form visible clusters or clumps even when healthy, while others remain more evenly distributed (see representative images in Figure 9). Although clustering does not necessarily indicate poor viability, excessive confluency in certain lines can lead to reduced health and increased cell death. Monitor cultures regularly and refer to Troubleshooting Problem 2 for guidance on when and how to split cells if needed.

***Note:*** Maintaining a strict every-other-day feeding schedule is less important than ensuring an appropriate cell density-to-media volume ratio. Earlier in differentiation (e.g., Day 30 or earlier), feeding every three days can be sufficient. However, additional media may be required depending on the proliferation rate and overall cell density.

**Figure 10:**
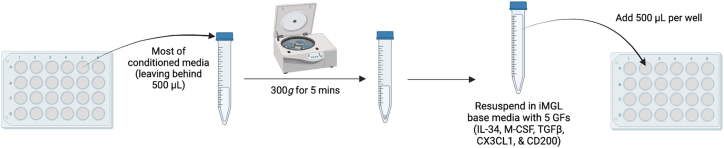
Workflow for Day 37 addition of CX3CL1 and CD200 to microglia cultures Schematic showing the workflow for supplementing microglia cultures with additional growth factors on Day 37. Most of the conditioned media is carefully removed from each well, leaving behind 500 μL to maintain paracrine signaling. Cells are collected, centrifuged at 300 × g for 5 min, and resuspended in fresh iMG base media supplemented with five growth factors: interleukin-34 (IL-34), macrophage colony-stimulating factor (M-CSF), transforming growth factor beta 1 (TGF-β1), fractalkine (CX3CL1), and CD200. The cell suspension is then added back at 500 μL per well. This general workflow can also be adapted for other desired treatments or media supplements throughout the culture duration.

4. **Day 17:** Incorporation of CX3CL1 and CD200 on Day 37 on iMG differentiation (see Figure 10).
  a. Carefully collect the media from each well, leaving 500 μL per well.
  b. Centrifuge the collected media at 300 × *g* for 5 min to pellet any non-adherent cells.
  c. Resuspend the pelleted cells in iMG base media supplemented with 100 ng/mL IL-34, 25 ng/mL M-CSF, 50 ng/mL TGF-β1, 200 ng/mL CX3CL1, and 200 ng/mL CD200.
  d. Add 0.5 mL of the resuspended cells back into the original wells.

***Note:*** On day 17, the concentration of CX3CL1 and CD200 is doubled to account for the 500 μL of old media remaining in each well. Subsequent feedings should use standard concentrations (100 ng/mL CX3CL1 and 100 ng/mL CD200).

5. **Day 19:** Feed Day 39 microglia with supplemented iMG base media.
  a. Add 0.5 mL iMG base media supplemented with 100 ng/mL IL-34, 25 ng/mL M-CSF, 50 ng/mL TGF-β1, 100 ng/mL CX3CL1, and 100 ng/mL CD200 to each well.

***Note:*** Do not remove any existing media prior to feeding.

***Note:*** Because microglia remain proliferative after Day 20, it is possible to plate fewer cells than the final number desired for triple culture assembly. However, the optimal plating density may vary depending on the cell line and should be determined empirically.

6. Microglia are ready for replating into triple culture between iMG differentiation Days 39 and 42.

### Thaw and differentiate D4 iPSC neurons

**Timing: 18 days**

Here, we outline the steps for thawing and differentiating Day 4 iPSC-derived neurons in preparation for integration into the triple culture system.

***Note:*** We recommend plating Day 4 neurons when microglia reach Day 21 of differentiation; however, this timing can be adjusted by a few days based on experimental needs.

7. Coat plate with Poly-L-ornithine and Laminin one day before plating.
  a. Thaw Poly-L-ornithine and laminin aliquots at room temperature (RT, 20°C–22°C).
  b. Prepare a coating solution in PBS containing 100 μg/mL Poly-L-ornithine and 5 μg/mL laminin.
  c. Add the coating solution to plates and incubate overnight (12–24 h), at 37°C.

***Note:*** Coated plates can be left in the 37°C incubator for up to 3 days before use.

8. **Day 2:** Coat Poly-L-ornithine and Laminin plates with Matrigel #354234 in DMEM/F12 (8.7 μg/cm^2^).
  a. Aspirate the Poly-O-Laminin solution and wash plates once with PBS.
  b. Aliquot the appropriate volume (e.g., 12 mL total to coat 1 × 24 well plate) of **cold** DMEM/F12.
  c. Retrieve Matrigel aliquots from the −20°C freezer.
  d. Resuspend frozen Matrigel into the **cold** DMEM/F12 by gentle trituration.
  e. Filter the solution through a 40 μm cell strainer.
  f. Coat the desired number of plates needed for the final triple culture experiment.
  g. Incubate plates for at least 30 min at 37°C, then wash once with PBS before plating cells.

***Note:*** While double coating with Poly-L-ornithine, Laminin and high-concentration Matrigel is not required, we have found that it improves neuron adherence and reduces peeling.

***Note:*** iNs are plated directly into their final format and will not be replaced.

9. **Day 2:** Thaw and plate day 4 neurons.
  a. Warm resuspension media: Neurobasal Medium (NBM) supplemented with 1:100 B27 and 10 μM ROCK inhibitor (Y-27632, ROCKi) to 37°C.
  b. Warm D4 plating media: NBM supplemented with 1:50 B27, 10 μM ROCKi, 10 ng/mL BDNF, 10 ng/mL CNTF, 10 ng/ml GDNF, 1–10 μg/mL puromycin, and 2 μg/mL doxycycline to 37°C.
    ***Note:*** Puromycin concentration should be optimized for each line.
  c. Remove cryovials from liquid nitrogen and immediately place into a bead or water bath.
  d. Thaw vials until just thawed (approximately 2 min maximum).
  e. Recover cells:
    i. Slowly add 1 mL of warmed resuspension media dropwise to each vial.
      ***Note:*** Always add media dropwise when collecting cells in DMSO.
    ii. Transfer the contents to a labeled 15 mL conical tube.
    iii. Rinse each cryotube with an additional 1 mL of resuspension media and transfer to the same conical tube.
    iv. Add 2–3 mL of resuspension media dropwise to the conical tube.
    v. Gently triturate to mix.
    vi. Take a 15 μL sample for cell counting.
      ***Note:*** To enhance cell recovery and viability, gently invert the conical tube a few times before centrifugation to evenly disperse the freezing media. This helps prevent the cryoprotectant from pooling around the cell pellet and may reduce cellular stress.
  f. Centrifuge cells at 200 × *g* for 5 min.
  g. Carefully remove the supernatant and resuspend the cell pellet in D4 plating media.
  h. Plate neurons at a density of approximately 53,000 cells/cm^2^, although this can be adjusted based on experimental needs.
    ***Note:*** With proper freezing and banking techniques, post-thaw viability should be comparable to, or slightly lower than, the original freeze. If a significant drop in viability is observed after thawing, assess whether the cells are suitable for continued use.
10. **Day 3:** Perform a full media change.
  a. Gently perform a full media change using NBM supplemented with 1:50 B27, 10 ng/mL BDNF, 10 ng/mL CNTF, 10 ng/mL GDNF, 1–10 μg/mL puromycin, and 2 μg/mL doxycycline.
11. **Days 4–19:** Feed every 3–4 days.
  a. Perform half media changes every 3–4 days (e.g., Monday/Thursday or Tuesday/Friday) with a ½ media change using NBM supplemented with 1:50 B27, 10 ng/mL BDNF, 10 ng/mL CNTF, 10 ng/mL GDNF, 1–10 μg/mL puromycin, and 2 μg/mL doxycycline.

***Note:*** It is recommended to discontinue doxycycline and puromycin treatment between Days 5–8; however, the optimal timing may vary depending on the cell line and should be determined empirically.

***Note:*** To minimize cell detachment or retraction, perform gentle partial media changes. Avoid touching the pipette tip to the bottom of the well and add fresh media dropwise along the side of the well.

12. Neurons will be ready for astrocyte replating to initiate co-culture between Days 18 and 22 of differentiation.

***Note:*** If using a cell line prone to developing unwanted proliferative cells in mature neuron cultures, or if visible proliferative cells are already present, cytosine β-D-arabinofuranoside (AraC) can be added to the media at a final concentration of 6.66–8 μM (resulting in 3.33–4 μM in the total well volume) during the first half-media change on Day 7 or 8, as described in problem 5.

### Thaw and differentiate D8 iPSC astrocytes

**Timing: 13 days**

Here, we outline the steps for thawing and differentiating Day 8 iPSC-derived astrocytes in preparation for integration into the triple culture system.

***Note:*** We recommend plating day 8 astrocytes when microglia have reached Day 27 and neurons have reached Day 10 of differentiation; however, this timing can be adjusted by a few days based on experimental needs.

13. **Day 8:** Coat plates with GFR Matrigel in DMEM/F12 (8.7 μg/cm^2^) as described above.
  a. Aliquot the appropriate volume (e.g., 12 mL total to coat 1 × 24 well plate) of **cold** DMEM/F12.
  b. Retrieve Matrigel aliquots from the −20°C freezer.
  c. Resuspend frozen Matrigel into **cold** DMEM/F12 by gentle trituration.
  d. Filter with the solution through a 40 μm cell strainer.
  e. Coat the desired number of plates.
    ***Note:*** Since astrocytes will be re-plated later for triple culture assembly, we recommend initially plating them in larger wells (e.g., 6-well plates or 10 cm dishes) to allow sufficient space for proper differentiation).
  f. Incubate plates for least 30 min at 37°C, then wash once with PBS before plating cells.
14. **Day 8:** Thaw and plate astrocytes.
  a. Warm resuspension media: FGF Base Media supplemented with 1:100 B27 and 10 μM ROCKi.
  b. Warm D8 plating media: FGF Base Media supplemented with 1:50 B27, 8 ng/mL FGF, 5 ng/mL CNTF, 10 ng/mL BMP4, 2.5 μg/mL doxycycline, and 10 μM ROCKi.
  c. Remove astrocyte cryovials from liquid nitrogen and immediately place into a bead or water bath.
  d. Thaw vials until just thawed (approximately 2 min maximum).
  e. Recover cells:
    i. Slowly add 500 μL of warmed resuspension media dropwise to each vial.
    ii. Transfer the contents to a labeled 15 mL conical tube.
    iii. Wash the cryotube with 1 mL of resuspension media and add it to the conical tube.
    iv. Add an additional 2–3 mL of resuspension media dropwise to the conical tube.
    v. Gently triturate the cell suspension.
    vi. Take 20 μL for cell counting.
      ***Note:*** To enhance cell recovery and viability, gently invert the conical tube a few times before centrifugation to evenly disperse the freezing media. This helps prevent the cryoprotectant from pooling around the cell pellet and may reduce cellular stress.
  f. Centrifuge cells at 220–300 × *g* for 5 min.
  g. Carefully remove the supernatant and resuspend the cell pellet in D8 plating media.
  h. Plate astrocytes at a density of approximately 45,000 cells/cm^2^, although this can be adjusted based on experimental needs.
    ***Note:*** With proper freezing and banking techniques, post-thaw viability should be comparable to, or slightly lower than, the original freeze. If a significant drop in viability is observed after thawing, assess whether the cells are suitable for continued use.
    **CRITICAL:** Astrocytes require sufficient space for proper differentiation. Astrocytes remain slightly proliferative between Days 8 and 12 of astrocyte differentiation while maintained in FGF media; thus, highly proliferative lines may require a lower initial plating density. If the average astrocyte cell size on Day 8 is smaller than 12 μm, increased proliferation during early differentiation may occur. Initial plating density should be optimized empirically for each cell line to ensure consistent final density.
15. **Day 9:** Perform a full media change.
  a. Perform a full media change using FGF Base Media supplemented with 1:50 B27, 8 ng/mL FGF, 5 ng/mL CNTF, 10 ng/mL BMP4, and 2.5 μg/mL doxycycline.
16. **Day 10–20:** Feed every 2–3 days.
  a. Perform half media changes every 2–3 days (e.g., Monday/Wednesday/Friday) using Maturation Base Media supplemented with 5 μg/mL N-acetylcysteine, 5 ng/mL heparin-binding EGF-like gf, 10 ng/mL CNTF, 10 ng/mL BMP4, 500 μg/mL dbcAMP, and 2.5 μg/mL doxycycline.

**CRITICAL:** Introduction of Maturation Media on Day 10 is essential to promote consistent astrocyte differentiation and to ensure reproducibility across experiments.

***Note:*** When replating astrocytes on Day 8 for triple culture assembly, plate approximately twice the number of cells needed for the final co-culture. Significant cell loss typically occurs during initial attachment and the subsequent replating process, resulting in roughly half of the initially plated cells remaining. Refer also to Troubleshooting Problem 3.

17. Astrocytes are ready for replating into triple culture between Days 18 and 22.

### Tri-culture assembly

**Timing: 1–2 weeks**

**Figure 11:**
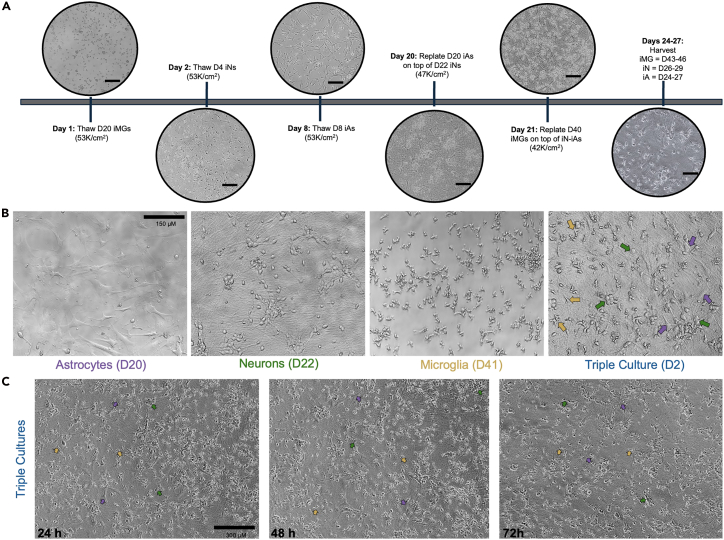
Schematic overview and representative images of iPSC-derived neuron-microglia-astrocyte triple culture assembly (A) Schematic overview of iPSC-derived neuron-microglia-astrocyte triple culture. The initial plating densities are noted. The days in bold refer to the start of thawing the first stock of cryopreserved cells and the non-bolded days refer to the day of differentiation for each individual cell type. Representative bright-field images depict cultures the day after each plating step (e.g., D20 microglia shown one day after thawing). Scale bars = 100 μM. (B) Representative 20× images of astrocytes, neurons, and microglia immediately prior to co-culture assembly, along with an example of triple culture morphology the day after microglia addition. Arrowheads highlight each cell type: yellow for microglia, green for neuronal somas, and purple for astrocytes. Astrocytes are typically less visible in triple culture under brightfield imaging as they are attached to the bottom of the well underneath the neuronal layer but can often be identified as bulges beneath neuronal layer. Scale bar = 150 μM. (C) Representative 4× images of triple cultures at 24, 48, and 72 h after microglia addition. Arrowheads again highlight neurons (green), astrocytes (purple), and microglia (yellow) over time. Scale bar = 300 μM.

Initially, cryopreserved stocks of each cell type are thawed and differentiated separately as described above. Astrocytes and microglia are then dissociated and replated on top of neuron cultures on neuron Day 22 and Day 23, respectively (Figure 11). The triple cultures are maintained for an additional three to twelve days. We provide an example timeline with differentiation days indicated for each cell type; however, the exact timing can be adjusted based on experimental needs. In general, we recommend that each cell type be nearly or fully differentiated prior to triple culture assembly, which is between Days 18–22 for neurons and astrocytes, and Days 39–42 for microglia. Representative images of cultures across the differentiation timeline are shown in Figure 11. Timing may also be tailored depending on the research question. For example, if assessing neuronal activity or connectivity, neurons may be further matured before initiating co-culture.

18. **Day 20:** Astrocyte Replating (Astrocyte D20, Neuron D22).
  a. Prepare replating media: Maturation Base Media supplemented with 5 μg/mL N-acetylcysteine, 5 ng/mL heparin-binding EGF-like GF, 10 ng/mL CNTF, 10 ng/mL BMP4, 500 μg/mL dbcAMP, 2.5 μg/mL doxycycline, 10 ng/mL GDNF, 10 ng/mL BDNF, 1:50 B27, and 10 μM ROCKi.
  b. Remove conditioned media from Day 20 astrocytes.
  c. Wash the 10 cm plate with 3–4 mL PBS.
  d. Add room temperature (20°C–22°C) diluted Accutase solution to the plate (1:3 Accutase: PBS + 10 μM ROCKi).
  e. Incubate at 37°C for 5–8 min, or until astrocytes lift off the plate.
  f. Collect the cells:
    i. Wash the plate with PBS to collect any remaining cells.
  g. Count the cells.
  h. Centrifuge cells at 220–300 × *g* for 5 min.
  i. Resuspend the cell pellet in the appropriate volume of replating media for direct plating onto neurons.
  j. Gently remove all media from neurons cultures.
  k. Plate D20 astrocytes directly on top of D22 neurons.
    ***Note:*** We typically replate astrocytes at a density of 47,000 cells/cm^2^; however, this can be adjusted depending on experimental needs.
    **CRITICAL:** Aged iNs are highly sensitive to peeling during full media changes. To minimize disruption, carefully remove the media and add fresh media with astrocytes dropwise. We recommend feeding one well at a time for better control and cell stability.
    ***Note:*** If astrocyte-neuron co-cultures (without microglia) are desired, we recommended continuing with Maturation Base Media supplemented with 5 μg/mL N-acetyl-cysteine, 5 ng/mL heparin-binding EGF-like GF, 10 ng/mL CNTF, 10 ng/mL BMP4, 500 μg/mL dbcAMP, 2.5 μg/mL doxycycline, 10 ng/mL GDNF, 10 ng/mL BDNF and 1:50 B27, rather than switching to the triple culture medium described below.
19. **Day 21:** Microglia Replating (Microglia D41, Astrocyte D21, Neuron D23).
  a. 24 h following astrocyte replating, add microglia to the astrocyte-neuron co-culture.
  b. Prepare triple culture media: BrainPhys (STEMCELL Technologies) supplemented with SM1 (STEMCELL Technologies), 25 ng/mL M-CSF, 100 ng/mL IL-34, 50 ng/mL TGF-β1, 100 ng/mL CX3CL1, and 100 ng/mL CD200.
  c. Collect conditioned media (containing floating cells) into conical tubes.
  d. Add 1 mL PBS to each well and incubate at room temperature (20°C–22°C) for 10–15 min.
  e. Transfer the PBS wash into the same conical tubes containing conditioned media.
  f. Inspect wells under a microscope to confirm all cells have detached. If cells remain, repeat steps d-e.
  g. Centrifuge cells at 220–300 × *g* for 5 min.
  h. Resuspend and combine all cell pellets in 1 mL triple culture media.
  i. Count the cells.
  j. Adjust the cell suspension to the appropriate volume with triple culture media for plating.
  k. Gently remove all media from astrocyte-neuron cultures.
  l. Plate Day 41 microglia directly onto Day 23 neurons and Day 21 astrocytes.

***Note:*** We typically replate microglia at a density of 42,000 cells/cm^2^; however, this can be adjusted depending on experimental needs.

**CRITICAL:** Aged iNs are highly sensitive to peeling during full media changes. To minimize disruption, carefully remove the media and add fresh media with microglia dropwise. We recommend feeding one well at a time for better control and cell stability.

20. Feed triple cultures every 3 days.
  a. Perform half media changes every 3 days using BrainPhys (STEMCELL Technologies) supplemented with SM1 (STEMCELL Technologies), 25 ng/mL M-CSF, 100 ng/mL IL-34, 50 ng/mL TGF-β1, 100 ng/mL CX3CL1, and 100 ng/mL CD200.
21. Use triple cultures for experimentation within 3–12 days after microglia addition.

***Note:*** Prior to collecting media for analysis, perform a full media change at least one media-change time point before harvest. For example, if planning to harvest on Day 6, perform a full media change 48 h earlier (on Day 4) to minimize residual factors in conditioned media.

**Figure 12:**
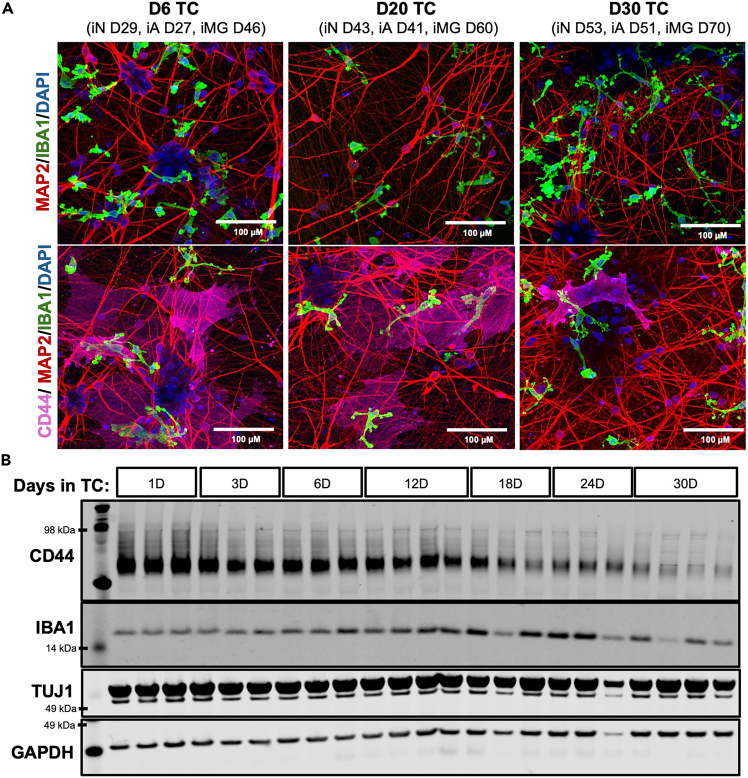
Longitudinal triple cultures (A) Representative immunostaining of triple cultures (iPSC line: BR33), featuring neurons (MAP2), microglia (IBA1), and astrocytes (CD44), maintained for 6, 20, or 30 days as indicated. Scale bar = 100 μM. (B) Representative Western blot of triple cultures collected at different time points, probed for CD44, IBA1, TUJ1, and GAPDH.

***Note:*** In most experiments, triple cultures have been maintained for up to 6 days following microglia addition. However, extended culture is possible, with microglia and neurons remaining stable for over 30 days. In contrast, astrocyte coverage typically begins to decline after approximately two weeks in triple culture (Figure 12), possibly due to substrate degradation or insufficient astrocyte growth factor support. For extended culture durations, we recommend optimizing coating conditions and considering the removal of microglia-specific growth factors and/or the addition of astrocyte-supportive factors commonly used in astrocyte maturation media (refer to Lish et al.^1^ for detailed media conditions tested in triple cultures).

## Expected outcomes

The protocols for differentiating iPSC-derived neurons, astrocytes, and microglia are highly robust and should consistently produce cultures with over 98% of cells expressing markers indicative of their respective cell types.^3^^,^^4^^,^^5^^,^^6^^,^^8^^,^^9^ Following six days in a triple-culture system, the cell population typically achieves an approximate ratio of 6:2:3 for neurons, astrocytes, and microglia.^1^^,^^2^ Importantly, the final cellular ratio differs from the initial seeding densities (52,000 cells/cm^2^ neurons, 47,000 cells/cm^2^ astrocytes, and 42,000 cells/cm^2^ microglia). Several factors contribute to this shift: neurons are plated on Day 4 of differentiation, when they are still relatively immature and retain minimal but some proliferative potential; astrocytes are seeded at a higher density to account for partial cell loss during replating and attachment; and microglia maintain limited proliferative capacity throughout the culture period. As a result, final cell ratios naturally diverge from the starting seeding densities, although this divergence can be predicted and accounted for to maintain consistent cell ratios across lines. Importantly, the triple culture medium described here is also compatible with monocultures and co-cultures of each individual cell type when differentiated according to the timelines outlined in this protocol, enabling direct comparisons of cellular phenotypes in the presence or absence of different cell types.^1^^,^^2^

Throughout protocol optimization, we observed that astrocyte morphology varied substantially depending on the culture medium. Astrocytes maintained in astrocyte maturation media retained a broader range of morphologies, including populations with extensive branching.^1^ In contrast, cultures maintained in other media formulations exhibited reduced morphological diversity. These findings are consistent with recent reports demonstrating that astrocytes derived from human glial-restricted progenitors using CNTF- or BMP4-based media develop distinct morphologies despite retaining mature astrocyte functions.^10^ Notably, these morphological patterns resemble those observed in our iAstrocyte cultures, with BMP4-driven astrocytes exhibiting more box-like morphologies and CNTF-driven astrocytes displaying elongated processes. Even partial replacement of maturation media with BrainPhys or other formulations reduced astrocyte heterogeneity.^1^ Collectively, these findings highlight the critical influence of culture conditions on astrocyte morphology and emphasize the importance of accounting for media differences when comparing results across experiments.

Single-cell RNA sequencing (scRNA-seq) and proteomic analysis of six-day triple cultures reveal distinct transcriptional and cellular responses to the shared environment.^1^ Microglia adopt a less inflammatory transcriptional profile and exhibit more ramified morphologies, while neurons demonstrate increased activity and enhanced dendritic spine density. Importantly, triple cultures remain responsive to inflammatory stimuli and can be adapted by altering cell-type configurations to interrogate how intercellular interactions influence cellular states and molecular pathways.

## Limitations

The co-culture modality described herein provides a unique and practical means of studying intercellular communication, yielding insights that are difficult to obtain from monocultures alone. Nonetheless, these models cannot fully replicate the complexity of *in vivo* systems. For instance, regional variations in cell type ratios and functional states, interactions with native extracellular matrix, and the involvement of additional cell types (e.g., inhibitory neurons, oligodendrocytes, pericytes, endothelial cells) remain unaccounted for in this system. Furthermore, most applications of this protocol have been conducted over relatively acute culture periods.^1^^,^^2^ However, the ability to use shorter co-cultures alongside cryopreserved intermediate cell types allows for high-throughput screening across various genetic backgrounds. This rapid and efficient strategy enables the identification of acutely emerging phenotypes, which can be additionally evaluated using other culture modalities, such as 3D cultures or organ on-a-chip systems. Finally, inter-sample variability can arise from differences in iPSC lines, differentiation efficiencies, and culture conditions. Rigorous quality control of cryopreserved stocks, including routine immunostaining for cell-type markers and functional assays, is therefore critical to ensure reproducibility and reliable interpretation of results.

## Troubleshooting

### Problem 1

Neurons detach from the plate during media changes (related to Step 18J and 19K in tri-culture assembly).

### Potential solution

• Media removal: Carefully remove media from the top of the well using a pipette, ensuring the pipette tip does not touch the bottom of the well where the cells are attached
• Media addition: Add fresh media dropwise along the side of the well to minimize direct disturbance to the cells. Avoid dispensing media directly onto the cells, as this can dislodge them.
• Electronic pipetting: Consider using an electronic pipette for media changes, as it provides more controlled dispensing and can help reduce mechanical stress when feeding delicate neurons.
• Full media change for mature neurons: For neurons that are more mature (e.g., day 20+), feed one well at a time to minimize the time cells are without media.
• Additional precautions: If neuron detachment persists, add fresh media to the side of the well opposite the area where detachment or “peeling” is occurring. This helps minimize mechanical stress and prevents further disruption to the affected region.

### Problem 2

**Figure 13:**
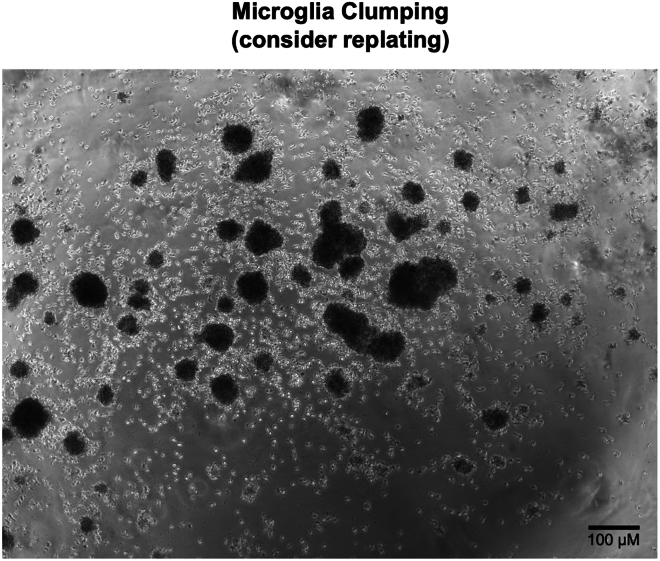
Representative image of excessive microglia clumping warranting consideration for replating Phase-contrast image showing a microglia culture with substantial clump formation, an example of a condition where replating is recommended to maintain microglia health (see troubleshootingproblem 2). Although some clumping is normal in certain iPSC lines, widespread and dense cluster formation can indicate overcrowding, which may impair cell viability and differentiation if not addressed. Scale bar = 100 μM.

Microglia proliferate excessively between days 20 – 37 of differentiation, leading to significant clumping and increased chance of cell death (related to Step 3 in thaw and differentiate D20 iPSC microglia). See Figure 13 for an example of a culture where the following interventions would be recommended.

### Potential solution

• Increase media volume: Add fresh media directly to the well without removing any existing media, bringing the total volume to 2 mL for a 24-well plate format. Maintaining an optimal cell density-to-media volume ratio is critical for microglia health.
• Replate overcrowded cells: If increasing the media volume does not resolve overcrowding within 24 h, replate the microglia into additional wells following the steps below (also see Figure 8):
• Coat phenol red-free Matrigel plates and warm fresh iMG base media.

***Note:*** For replating, prepare a 1:1 mixture of conditioned media (collected from the original culture) and fresh iMG base media supplemented with 100 ng/mL IL-34, 25 ng/mL M-CSF, and 50 ng/mL TGF-β1. Retaining conditioned media is essential, as microglia secrete paracrine factors that are critical for continued differentiation.

• Collect conditioned media (containing floating cells) into conical tubes.
• Add 1 mL PBS to each well and incubate at room temperature (20°C–22°C) for 5–10 min.
• Transfer the PBS wash to separate conical tubes, keeping PBS separate from the conditioned media.
• Inspect wells under a microscope to confirm all cells have detached. If necessary, repeat PBS washes.
• Centrifuge all tubes at 220–300 × *g* for 5 min.
• Carefully remove the supernatant, saving the conditioned media. Resuspend and combine cell pellets in 1 mL of conditioned media.
• Count the cells.
• Adjust the final suspension volume using a 1:1 mixture of conditioned media and fresh supplemented iMG base media.
• Plate 80,000–120,000 cells per well in 1 mL of media (24-well plate format).
• Two days after replating, perform a media change following **Day 22** of thaw and differentiate D20 iPSC microglia*,* and then continue with one-third media changes as scheduled.

### Problem 3

Low astrocyte recovery during replating, resulting in insufficient numbers for co-culture (related to Step 18 in tri-culture assembly).

### Potential solution

• Increase initial thawing: Thaw 2–3 times more immature astrocytes on Day 8 of the differentiation protocol to account for expected cell loss during initial plating and replating.
• Optimize dissociation timing: Perform dissociation for exactly 5 min at 37°C using 1:3 diluted Accutase in PBS. Longer incubation times can compromise astrocyte viability.
• Gently wash cells post-dissociation: After incubation, gently wash cells off the plate using an equal volume of PBS. Astrocytes at this stage are particularly fragile and susceptible to mechanical stress.
• Centrifuge under optimized conditions: Centrifuge cells at 500 × *g* for 5 min using a swinging bucket centrifuge to maximize cell recovery.
• Use appropriate tubes: Perform centrifugation in 5 mL Eppendorf microcentrifuge tubes (Sigma, #EP0030119487) to improve pellet formation and minimize cell loss.
• Pre-coat tubes to prevent cell loss: Pre-coat collection tubes with anti-adherence rinsing solution (STEMCELL Technologies, #07010) before adding the cell suspension to minimize astrocyte adhesion to tube walls.

### Problem 4

Microglia are lost and/or neurons peel during fixation at the experimental endpoint.

### Potential solution

• Perform a gentle pre-fixation step: Without removing the conditioned media, carefully spike in 4% PFA at a 1:10 dilution (e.g., if the culture well contains 180 μL of media, add 20 μL of 4% PFA) to achieve a 0.4% PFA concentration. Return the plates to the incubator at 37°C for 20 min to allow gradual fixation, stabilizing the microglia. After incubation, gently remove the media and proceed with standard fixation by adding 4% PFA and incubating at room temperature (20°C–22°C) for 15 min. This pre-fixation step helps anchor microglia in place, minimizing cell displacement during media removal and prevents peeling of the neuronal cell layer.
• Alternative fixation with sucrose supplementation: Without removing the conditioned media, carefully spike in freshly prepared 8% PFA supplemented with 8% sucrose. Add an equal volume of 8% PFA + 8% sucrose solution to the media already present in the well. This results in a final concentration of 4% PFA and 4% sucrose. For example, if a well contains 180 μL of media, add 180 μL of 8% PFA + 8% sucrose solution. Sucrose acts as an osmoprotectant, helping to maintain neuronal structure and minimize swelling or shrinkage during fixation. By adding the fixative directly to the wells, this can prevent peeling and cell loss during media removal.
• Use PBS containing magnesium and calcium. During fixation, use PBS supplemented with magnesium and calcium. This helps preserve neuronal integrity and reduces the risk of the neuronal layer peeling.
• Minimize mechanical disturbance: Use slow and gentle pipetting when removing media or adding fixative to avoid dislodging cells. An electronic pipette can further improve consistency and minimize mechanical stress.

### Problem 5

**Figure 14:**
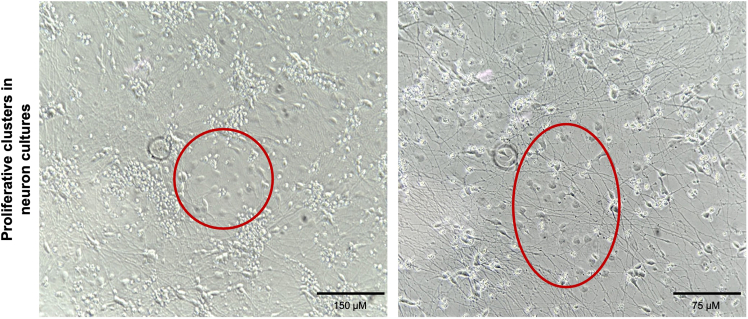
Representative images of proliferative cell clusters emerging in neuron cultures Phase-contrast images showing examples of unwanted proliferative clusters (highlighted with red circles) in iPSC-derived neuron cultures (see problem 5). If observed, optimization of puromycin concentration and/or treatment with cytosine β-D-arabinofuranoside (AraC) is recommended to selectively eliminate dividing cells while preserving neuron health. Scale bar = 150 μM.

Proliferative cells appear in neuron cultures during days 4 to 21 of differentiation (see Figure 14).

### Potential solution

• Optimize puromycin concentration: Puromycin selection may need to be fine-tuned for each iPSC line to effectively eliminate proliferative cells without harming differentiating neurons.
• Incorporate Cytosine β-D-arabinofuranoside (AraC): Add AraC during the first half-media change (e.g., on Day 7 or 8) to selectively inhibit DNA synthesis in dividing cells and prevent expansion of unwanted proliferative populations. AraC should be added to the fresh media at a concentration of 6.66–8 μM, resulting in a final concentration of 3.33–4 μM in the total media volume in the well after mixing with the existing media. If proliferative cells persist, a second AraC treatment can be administered during the next half-media change at the same concentration; however, it is generally recommended to limit AraC exposure to a single half-media change to minimize potential neuronal toxicity.

***Note:*** Although short-term AraC exposure at these concentrations has not been shown to adversely affect mature neurons, for consistency, we recommend treating all experimentally paired cell lines with identical AraC concentrations and timing.

### Problem 6

Proliferative cells appear in astrocyte cultures during days 8 to 21 of differentiation.

### Potential solution

• Optimize puromycin and hygromycin concentration: Puromycin and/or hygromycin selection may need to be fine-tuned for each iPSC line to effectively eliminate proliferative cells without harming differentiating astrocytes.
• Optimize seeding density: Seeding density is critical for successful astrocyte differentiation and must be tailored to each iPSC line due to varying proliferation rates. For Day −1 (related to step 13 under Generating and banking D8 induced astrocytes from SOX9 and NFIB iPSCs), test a range of 100,000 to 200,000 cells/cm^2^ to ensure optimal starting density for differentiation. For day 8 seeding, test a range of 30,000 to 80,000 cells/cm^2^ to support continued maturation and prevent overcrowding. Overcrowded astrocytes lack sufficient space to differentiate properly, leading to reduced maturation and increased proliferation.
• Monitor cell maturity: When freezing cell stocks on day 8, assess the average cell size, which should be greater than 13 μM. If the average size is below this, it may indicate immature cells with a higher tendency to proliferate, particularly during early differentiation (days 8–12) when FBS is still present in the media. In these cases, seeding day 8 astrocytes at a lower density may help promote proper maturation.

### Problem 7

Low yield of D4 iNs or D8 iAs due to increased cell death during puromycin or hygromycin selection during differentiation.

### Potential solution

• Monitor number of passages since transduction: Bank several vials of transduced iPSCs soon after transduction to maintain a low passage stock. Attempt differentiation with a lower passage vial.
• Re-transduce iPSCs: Failure to express sufficient antibiotic resistance may indicate insufficient levels of construct in the iPSC line. Lentivirus concentration can be optimized with a trial using 0.5× - 4× of previous MOI, or 0.5μL/380K cells - 4μL/380K cells.
• Evaluate transduction efficiency: Co-transduce iPSCs with a marker construct (ex: GFP) in addition to NGN2 and rtTA constructs. Observe transduction efficiency 1–2 days after transduction by fluorescence (if the marker construct is also doxycycline-inducible, add doxycycline 24 h after transduction and evaluate efficiency 48 h after transduction). Co-transduction rate is generally high; presence of marker plasmid should reliably suggest presence of co-transduced plasmids. If transduction efficiency is less than 70%, consider re-transduction.
• Optimize puromycin/hygromycin concentration: if continued cell death during differentiation in the absence of morphology issues or persistent proliferative cell types, optimize/decrease puromycin or hygromycin concentration during differentiation.

## Resource availability

### Lead contact

Further information and requests for resources and reagents should be directed to and will be fulfilled by the lead contact, Tracy Young-Pearse (tpearse@bwh.harvard.edu).

### Technical contact

Technical questions on executing this protocol should be directed to and will be answered by the technical contact, Christina R. Muratore (cmuratore@bwh.harvard.edu).

### Materials availability

This study did not generate new, unique reagents.

### Data and code availability

This study did not generate/analyze new datasets or code.

## Acknowledgments

We thank the AMP-AD consortium for valuable feedback and data sharing (see also “data and code availability”); the NeuroTechnology Studio at Brigham and Women’s Hospital for providing Zeiss LSM710, Andor Dragonfly 600 Spinning Disk, and Zeiss LSM880 + Fast Airyscan confocal instrument access and consultation on data acquisition and data analysis; iPSC NeuroHub at Brigham and Women’s Hospital for technical assistance; Maria Lehtinen, Vik Khurana, and Lee Rubin for their Dissertation Advisory Committee guidance; and the members of the Young-Pearse lab for their input. This work was supported by the National Science Foundation Graduate Research Fellowship under grant no. 1745303 and by NIH grants U54AG090669, U01AG061356, RF1AG057473, and U01AG046152 as part of the AMP-AD consortium, as well as NIH grants R01AG066831, RF1AG072167, U01AG072572, R01NS117446, R01AG055909, U54AG090669, and R01NS142017. This work was also supported by an Alzheimer’s Association Zenith Award (T.L.Y.-P.). Scientific illustrations in this work incorporate elements adapted from BioRender.

## Author contributions

Conceptualization, A.M.L. and T.L.Y.-P.; methodology, A.M.L., N.A., C.R.M., and T.L.Y.-P.; validation, A.M.L., P.C.G., G.A.O., N.A., A.J.C., and S.E.H.; formal analysis, A.M.L. and T.L.Y.-P.; investigation, A.M.L., N.A., and T.L.Y.-P.; resources, T.L.Y.-P.; data curation, A.M.L., P.C.G., S.E.H., A.J.C., and T.L.Y.-P.; writing – original draft, A.M.L. and T.L.Y.-P.; writing – review and editing, A.M.L., N.A., G.A.O., P.C.G., C.R.M., A.J.C., S.E.H., and T.L.Y.-P.; visualization, A.M.L., P.C.G., A.J.C., and T.L.Y.-P.; supervision, T.L.Y.-P.; project administration, T.L.Y.-P.; funding acquisition, T.L.Y.-P. and A.M.L.

## Declaration of interests

The authors declare no competing interests.
